# Functional metagenomic approach to identify overlooked antibiotic resistance mutations in bacterial rRNA

**DOI:** 10.1038/s41598-018-23474-4

**Published:** 2018-04-03

**Authors:** Kentaro Miyazaki, Kei Kitahara

**Affiliations:** 1 Department of Life Science and Biotechnology, Bioproduction Research Institute, National Institute of Advanced Industrial Science and Technology (AIST), Central 6, 1-1-1 Higashi, Tsukuba Ibaraki, 305-8566 Japan; 2 Department of Computational Biology and Medical Sciences, Graduate School of Frontier Sciences, The University of Tokyo, 5-1-5 Kashiwanoha, Kashiwa, Chiba, 277-8561 Japan; 3 Ambitious Leader’s Program, Graduate School of Science, Hokkaido University, N10W8, Kita-ku, Sapporo, 060-0810 Hokkaido, Japan; 4 Department of Chemistry, Graduate School of Science, Hokkaido University, N10W8, Kita-ku, Sapporo, 060-0810 Hokkaido, Japan

## Abstract

Our knowledge as to how bacteria acquire antibiotic resistance is still fragmented, especially for the ribosome-targeting drugs. In this study, with the aim of finding novel mechanisms that render bacteria resistant to the ribosome-targeting antibiotics, we developed a general method to systematically screen for antibiotic resistant 16 S ribosomal RNAs (rRNAs), which are the major target for multiple antibiotics (e.g. spectinomycin, tetracycline, and aminoglycosides), and identify point mutations therein. We used *Escherichia coli* ∆7, a null mutant of the *rrn* (ribosomal RNA) operons, as a surrogate host organism to construct a metagenomic library of 16 S rRNA genes from the natural (non-clinical) environment. The library was screened for spectinomycin resistance to obtain four resistant 16 S rRNA genes from non-*E. coli* bacterial species. Bioinformatic analysis and site-directed mutagenesis identified three novel mutations - U1183C (the first mutation discovered in a region other than helix 34), and C1063U and U1189C in helix 34 - as well as three well-described mutations (C1066U, C1192G, and G1193A). These results strongly suggest that uncharacterized antibiotic resistance mutations still exist, even for traditional antibiotics.

## Introduction

Antibiotic resistance is a serious problem for human beings because pathogenic microorganisms that acquire such resistance void antibiotic treatments. Therefore, a tremendous effort has been made by researchers to identify specific resistance mechanisms and mutations that render bacteria resistant to antibiotics. These studies are beneficial for the timely detection and early diagnosis of resistant bacteria, which is key to prevent the spread of unwanted infectious diseases.

There are three main mechanisms for microorganisms to acquire antibiotic resistance: (i) enzymatic inactivation or modification of antibiotics (e.g. β-lactamases inactivate penicillin antibiotics)^1^; (ii) acquisition of mutation(s) in target sites of the antibiotics; and (iii) decreasing the net drug concentration in the cell by reducing drug permeability via cell wall or by increasing the activity of efflux pumps (e.g. tetracycline resistance)^2^. Among these, the second mechanism is often observed for ribosome-targeting drugs such as spectinomycin (Spc), aminoglycosides (e.g. kanamycin, streptomycin, neomycin), tetracycline, chloramphenicol, macrolides, lincomycins, streptogramin A, and oxazolidinones; the former three are known to target the 30 S subunit that contains the 16 S rRNA as its main component, whereas the others are known to attack the 50 S subunit that contains the 23 S rRNA as its main component^3^.

As described above, there are a large number of antibiotics that target the ribosome. This is because ribosomes play an essential role in protein biosynthesis, translating messenger RNA-encoded genetic information into proteins, which consists of sequential multistep reactions - initiation, elongation, termination, and recycling. Owing to these extremely elaborate reaction dynamics, there are different kinds of inhibitors targeting each step of the translation process^3–5^. As the ribosome is RNA-rich, and functionally critical sites exist mainly on RNAs (the decoding centre in 16 S rRNA and peptidyl transferase centre in 23 S rRNA), many antibiotic target sites exist on rRNAs, as do several resistant point mutations, accordingly^3,6,7^.

Researchers have long tried to list as many resistant point mutations in rRNAs as possible, by means of classical genetic experiments using organisms such as *Escherichia coli*^8^, *Halobacterium halobium*^9^, and *Mycoplasma smegmatis* (*rrn*^*−*^)^10^. There are, however, unavoidable drawbacks in these systems. In the *E. coli* system, the organism has as many as seven rRNA operons (*rrn* operons) in its genome. Owing to the high background derived from the endogenous (wild-type) rRNA genes, it is necessary to use a high copy number vector to characterize the function (i.e. antibiotic susceptibility) of mutant 16 S rRNA genes *in vivo*^8,11,12^. However, the handling of such non-simple genetic systems suffers from various technical difficulties, as pointed out previously^13^. Although *H. halobium* and *M. smegmatis* only have one *rrn* operon in their genome, and thus can partly solve the underlying problem in the *E. coli* system, they only show slow growth phenotypes and therefore, make it difficult to conduct reliable genetic experiments. It is thus uncertain whether all possible resistant mutations to an antibiotic have successfully and correctly been listed using these systems. It should be noted that *Thermus thermophilus*, a thermophilic strain with a single *rrn* operon, has been used for similar purposes i.e. to generate interesting insights on antibiotic resistance mutations^14,15^.

Here, we propose a new approach to circumvent these methodological problems in a simple way; we use *E. coli* Δ7, a null mutant of the *rrn* operon^16^, as a surrogate host organism and screen for the antibiotic resistance of various species’ rRNA genes, which are retrieved from environmental metagenomes. Admittedly, the use of the null mutant *E. coli* strain is not a novel approach and there are in fact some studies that use the strain to determine antibiotic resistance mutations in rRNAs^17,18^. The originality in our study resides in the methodology i.e. we use metagenomic rRNA genes that are directly extracted from the environment, while *E. coli* Δ7 is simply used as a surrogate organism that can be handled easily as a model microorganism. The rationale of our approach using the combination of non-*E. coli* rRNA genes and the *E. coli* host is based on our recent finding that various 16 S rRNAs, including those from a different class^19^ or a phylum^20^, are functionally compatible with the *E. coli* ribosome. We can genetically characterize the functions of a diverse array of heterologous 16 S rRNAs using *E. coli* Δ7 as a common platform^20^. In this study, we applied this technique, named Comparative RNA Function Analysis^20^, to test whether we can find novel and biologically relevant antibiotic resistance mutations. Specifically, we used a traditional antibiotic, Spc, mutations to which are supposed to have been thoroughly investigated, as a model antibiotic. A metagenomic library of non-clinical environments, which are considered to be a reservoir of antibiotic resistance^21–23^, was constructed using *E. coli* Δ7 as a host, then functionally screened for Spc resistance. As the result, we successfully obtained four 16 S rRNA genes from non-*E. coli* bacterial species carrying Spc resistance mutations. Further analysis of these genes revealed that three point mutations (C1063U, U1183C, and U1189C, *E. coli* numbering), which have not been, to our best knowledge, reported in any literature thus far, render bacteria resistant to Spc. Our results strongly suggest that there are many unfound and therefore uncharacterized antibiotic resistance point mutations in 16 S rRNA genes in natural environments, and also possibly in clinical environments.

## Results

### Metagenomic library screening for Spc resistant 16 S rRNA genes

To construct a metagenomic library of 16 S rRNA genes, we prepared a mixture of metagenomic samples directly extracted from natural environments (fermentation products, activated sludge, and wood compost). The full-length 16 S rRNA genes were PCR-amplified from the metagenomic mixture using a set of universal primers^24^ and the amplicon was cloned in the expression vector pMY205mPAG2 (encoding trimethoprim [Tmp] resistance gene, p15A ori, and *E. coli rrnB* operon containing a 16 S rRNA gene) by replacing the pre-existing *E. coli* 16 S rRNA gene in the vector with the amplicon^24^. An *E. coli* ∆7 strain (MY101), a null mutant of the *rrn* operons in the genome, was then transformed with the plasmid library and screened for functionally compatible heterologous 16 S rRNA genes based on the ability to support the growth of the host (in the absence of Spc). MY205 colonies that appeared on the LB/Tmp agar plates containing sucrose (approximately 2,000) were then secondarily screened on LB/Tmp agar plates containing 40 µg/mL Spc. Four Spc resistant clones were selected, which were named NHMcSpc1, mgSpc1, mgSpc2, and mgSpc5.

**Figure 1:**
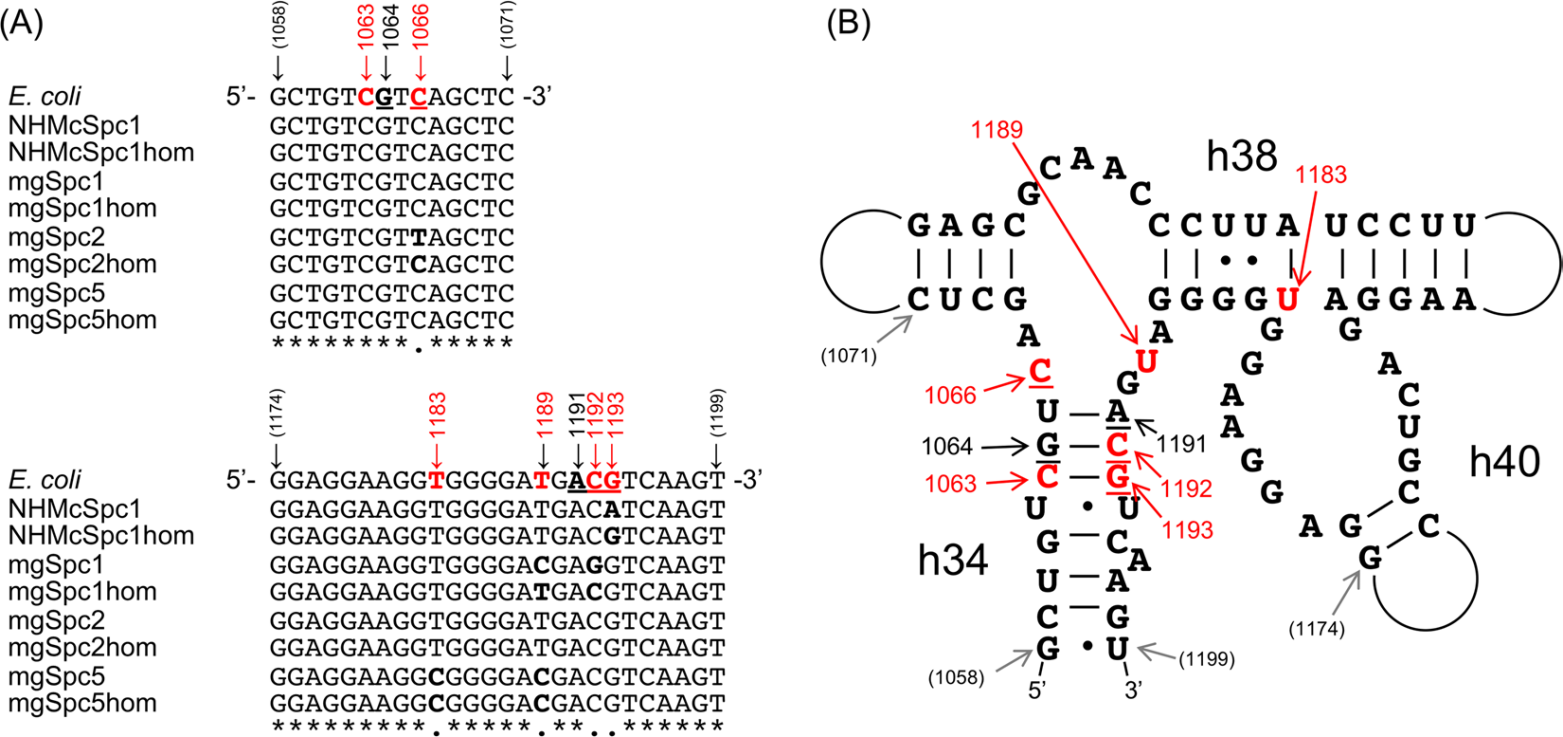
Putative spectinomycin (Spc) resistance point mutations in 16 S rRNA gene as inferred by Spc resistant 16 S rRNA genes from the metagenome. (**A**) Multiple sequence alignment of 16 S rRNA genes from *E. coli*, metagenomically retrieved Spc resistant clones (NHMcSpc1, mgSpc1, mgSpc2, and mgSpc5), and their closest homologues (suffixed “hom” to its parent’s name) from the NCBI database. Known resistance mutation sites are underlined in the *E. coli* sequence and putative resistance nucleotides to Spc are highlighted in red. Sites that share identical nucleotides among all sequences are shown with asterisks and those not completely conserved are shown with a dot below the alignment. (**B**) Secondary structure of *E. coli* 16 S rRNA around the Spc resistance mutation sites aligned in (**A**). Putative resistance nucleotides to Spc are coloured in red.

Table 1 summarizes the sequence properties of the resistant 16 S rRNA genes. All the genes were derived from Gammaproteobacteria, which showed 82–96% sequence identities to the *E. coli* 16 S rRNA (*rrnB*) gene. Each gene sequence was used as a query to BLAST-search for the closest homologues in the NCBI database; sequence comparison between the queries and the homologues, as well as *E. coli* sequence (Spc susceptible) would be effective for predicting resistance mutations. Figure 1A shows the sequence alignment of the two hot spots (G1058 to G1071 and G1174 to U1199, *E*. *coli* numbering) where Spc resistance mutations have been frequently discovered (G1064, C1066, A1191, C1192, and G1193, underlined in the *E. coli* sequence)^25–34^. The secondary structure of 16 S rRNA, including the hot spots, is shown in Fig. 1B. Putative resistance mutations identified in the metagenomically retrieved resistance genes are highlighted in red in Fig. 1A,B and summarized in Table 2. U1183C and U1189C mutations have not been described as Spc resistance mutations, but our sequence analysis (Fig. 1A) suggested that these mutations might be involved in resistance. Our approach to confirm the effect of these putative resistance mutations included: (i) changing the putative resistance mutations in the metagenomically-retrieved resistant genes to the non-resistant type nucleotides (*E. coli* sequence as a reference), either individually or in combination with other nucleotides and (ii) introducing the putative resistance mutations into the *E. coli* 16 S rRNA gene. The constructed mutant 16 S rRNA genes were then introduced into *E. coli* Δ7 and the resistance was tested in the presence of various concentrations of Spc.

**Table 1:** Spectinomycin (Spc)-resistant 16 S rRNA genes retrieved from the metagenome and their closest homologues.

Clone (Accession ID)	Closest homologues				Identity to *E. coli* 16 S rRNA (*rrnB*)
	Accession ID	Strain	Phylogeny (phylum; class; order; family)	Identity	
NHMcSpc1 (LC306682)	CP017802.1	*Raoultella ornithinolytica* strain MG	Proteobacteria; Gammaproteobacteria; Enterobacterales; Enterobacteriaceae	99% (1536/1540)	96% (1484/1542)
mgSpc1 (LC306679)	NR_074692.1	*Thioalkalivibrio sulfidophilus* strain HL-EbGR7	Proteobacteria; Gammaproteobacteria; Chromatiales; Ectothiorhodospiraceae	92% (1418/1549)	83% (1302/1558)
mgSpc2 (LC306680)	NR_145539.1	*Chujaibacter soli* strain KIS55–21	Proteobacteria; Gammaproteobacteria; Xanthomonadales; Xanthomonadaceae	99% (1466/1486)	82% (1286/1550)
mgSpc5 (LC306681)	NR_108606.1	*Thalassolituus marinus* strain IMCC1826	Proteobacteria; Gammaproteobacteria; Oceanospirillales; Oceanospirillaceae	99% (1464/1472)	86% (1330/1542)

**Table 2:** Putative resistance mutations in the Spectinomycin (Spc)-resistant 16 S rRNA genes retrieved from the metagenome.

Clone	Putative resistance mutations (helix number)
NHMcSpc1	G1193A (helix 34)
mgSpc1	U1189C (helix 34), C1192G (helix 34)
mgSpc2	C1066U (helix 34)
mgSpc5	U1183C (helix 38), U1189C (helix 34)

### Mutation study of the putative Spc resistance mutations in the metagenomically retrieved 16 S rRNA genes

*E. coli* MY205 derivatives were grown in LB broth containing various concentrations (0–1,024 µg/mL) of Spc and the growth phenotypes (growth curves drawn from OD_600_ values) were monitored for 10 h. The wild-type *E. coli* (MY205 complemented by self 16 S rRNA gene from *rrnB*) had a minimum inhibitory concentration (MIC) of 32 µg/mL in this system (Table 3, Supplementary Fig. S1a). Similar analysis was conducted for each metagenomic 16 S rRNA clone (Supplementary Fig. S1b–S1e) and the MICs were determined as summarized in Table 4.

**Table 3:** Minimal inhibitory concentrations (MICs) of Spectinomycin (Spc) for *E. coli* MY205 harbouring wild-type or mutated *E coli* 16 S rRNA gene.

Mutations	MIC, µg/mL	Notes
None	32	Wild-type 16 S rRNA gene of *E. coli*
C1063U	256	Novel resistance mutation (helix 34) forming a stable (non-Watson–Crick) U–G base pair (U1063–G1193)
C1066U	>1,024	A mutation known to confer high level resistance in *E. coli*^23^
U1183C	1024	Novel resistance mutation (helix 38)
U1189C	256	Novel resistance mutation (helix 34)
U1183C/U1189C	256	Double mutant
C1192G	>1,024	A mutation known to confer high-level resistance in *E. coli*^22^
U1189C/C1192G	>1,024	Double mutant
G1193A	>1,024	A mutation known to confer low-level resistance in the chloroplast^21,31^ forming an unstable base pair (C1063–A1193)
C1063U/G1193A	1024	Double mutant forming a stable Watson–Crick base pair (U1063–A1193)

**Table 4:** Minimal inhibitory concentrations (MICs) of Spectinomycin (Spc) for *E. coli* MY205 derivatives harbouring the 16 S rRNA genes retrieved from the metagenome.

16 S rRNA genes	Mutations	MIC, µg/mL	Notes
NHMcSpc1	None (Wild-type)	>1,024	Harbouring the putative resistance mutation G1193A (in helix 34) forming an unstable base pair (C1063–A1193)
	C1063U	512	Introduces a stable Watson–Crick base pair (U1063–A1193)
	A1193G	32	Introduces a *E. coli*-type Watson–Crick base pair (C1063–G1193)
	C1063U/A1193G	256	Introduces a stable (non-Watson–Crick) U–G base pair (U1063–G1193)
mgSpc1	None (Wild-type)	>1,024	Contains the putative resistance mutations U1189C (helix 34) and C1192G (helix 34)
	C1189U	>1,024	Reverts to the putative resistance nucleotide C1189 in *E. coli* type (U)
	G1192C	256	Reverts to the putative resistance nucleotide G1192 in *E. coli* type (C)
	C1189U/G1192C	64	Double mutant
mgSpc2	None (Wild-type)	1,024	Contains the putative resistance mutation C1066U (helix 34)
	U1066C	256	Reverts to the putative resistance nucleotide U1066 in *E. coli* type (C)
mgSpc5	None (Wild-type)	256	Contains the putative resistance mutations U1183C (helix 38) and U1189C (helix 34)
	C1183U	512	Reverts to the putative resistance nucleotide C1183 in *E. coli* type (U)
	C1189U	512	Reverts to the putative resistance nucleotide C1189 in *E. coli* type (U)
	C1183U/C1189U	128	Double mutant

MY205 with NHMcSpc1 had high-level Spc resistance with MIC of >1,024 µg/mL (Table 4, Supplementary Fig. S1b). Within this gene, a putative resistance mutation, G1193A, was inferred (Fig. 1A, Table 2), which is known to confer a low-level resistance in plant chloroplasts^25,35^. As G1193 forms a Watson-Crick base pair with C1063 (C1063–G1193) in its homologue (NHMcSpc1hom) and also in the *E. coli* 16 S rRNA (Fig. 1), we considered that incorrect pairing of these bases might be related to the expression of Spc resistance, as well as their base-identities. Interestingly, by introducing a single point mutation, C1063U, in NHMcSpc1 to introduce a U-A Watson-Crick base pair (U1063-A1193), we found that Spc resistance was slightly (but significantly) reduced (MIC = 512 µg/mL) (Table 4). Additional introduction of G in the position 1093, which is expected to form a stable U-G base pair (U1063-G1193)^36^ was also effective in significantly reducing resistance (MIC = 256 µg/mL) (Table 4). When A1193 was reverted to G (C1063-G1193), the resistance was completely eradicated (MIC = 32 µg/mL) (Table 4). These results suggest that the mode of base-pairing between nucleotides 1063 and 1193 as well as the base identity at position 1193 determines whether a given 16 S rRNA sequence expresses Spc resistance.

MY205 with mgSpc1 showed a high-level resistance (MIC >1,024 µg/mL) (Table 4, Supplementary Fig. 1C) in which two putative resistance mutations, U1189C and C1192G, were inferred (Fig. 1A, Table 2). These bases in mgSpc1 were singly or doubly reverted to the nucleotides used in *E. coli* or mgSpc1hom. C1189U alone did not significantly alter the resistance (MIC >1,024 µg/mL) (Table 4), whereas G1192C largely reduced the resistance (MIC = 256 µg/mL) (Table 4). Their combination further reduced the resistance (MIC = 64 µg/mL) (Table 4), suggesting that G1192 is the major determinant and C1189 is a minor determinant for Spc resistance.

MY205 with mgSpc2 showed a high-level resistance (MIC = 1,024 µg/mL) (Table 4, Supplementary Fig. S1d), in which a single putative resistance mutation C1066U was inferred (Fig. 1A, Table 2). Reverting the U1066 nucleotide to the nucleotide used both in *E. coli* 16 S rRNA and mgSpc2hom (C) largely reduced the resistance (MIC = 256 µg/mL) (Table 4), verifying that the C1066U mutation was the key determinant in mgSpc2 for Spc resistance, as reported in many Spc resistant bacterial 16 S rRNAs^27^.

MY205 with mgSpc5 showed a mild but significant resistance (MIC = 256 µg/mL) (Table 4, Supplementary Fig. S1e). One unique characteristic in this gene is that the closest homologue mgSpc5hom (i.e. *T. marinus* strain IMCC1826) also shared the putative resistance mutations, U1183C and U1189C (Fig. 1A, Table 2). Both the C1183U and C1189U reverting mutations did not significantly reduce the resistance of mgSpc5, respectively (Table 4), whereas the double mutations (C1183U/C1189U) in the same gene slightly but significantly reduced the resistance (MIC = 128 µg/mL) (Table 4), suggesting that the simultaneous mutation of both nucleotides is the determinant of the Spc resistance in mgSpc5.

### Introducing the putative Spc resistance mutations into the *E. coli* 16 S rRNA gene

In the above subheading, we introduced reverting point mutations in metagenomically-retrieved 16 S rRNA genes to confirm that the putative resistance mutations we predicted in Table 2 were involved in Spc resistance (according to the first approach). During this process, we also addressed the significance of the mode of base-pairing between nucleotides 1063 and 1193 with respect to the expression of Spc resistance. In this subheading, we report the results of our second approach, in which the putative resistance mutations were introduced into the *E. coli* 16 S rRNA gene (*rrsB*). The effect of the mode of base-pairing between nucleotides 1063 and 1193 was also elucidated using the same *E. coli* 16 S rRNA gene.

In MY205, introducing G1193A mutation (found in NHMcSpc1) in *E. coli* 16 S rRNA gene conferred high-level resistance (MIC >1,024 µg/mL, Table 3, Supplementary Fig. S2a), confirming that this mutation renders bacterial 16 S rRNA highly resistant to Spc. The effect of G1193A, however, was slightly diminished when combined with C1063U mutation (U1063-A1193) (MIC = 1,024 µg/mL, Table 3, Supplementary Fig. S2a). C1063U single mutation (U1063–G1193) conferred a modest level of resistance (MIC = 256 µg/mL, Supplementary Fig. S2a). Collectively, strong base-pairing between nucleotides 1063 and 1193 seems to be an important factor in determining Spc susceptibility of the bacterial ribosome; C-A unstable base pair makes 16 S rRNA highly resistant to Spc while U-A and U-G stable base pairs at least partially reduce the Spc resistance compared to the C-A pair, although the U-A and U-G pairs certainly make 16 S rRNA less susceptible to Spc, compared to the cognate C-G pair (the strongest Watson-Crick base pair), respectively.

When U1189C (found in mgSpc1) was introduced into the *E. coli* 16 S rRNA gene, it rendered MY205 modestly resistant to Spc (MIC = 256 µg/mL) (Table 3, Supplementary Fig. S2b), whereas very high-level resistance (MIC >1,024 µg/mL) was conferred by C1192G (also found in mgSpc1) mutation (Table 3, Supplementary Fig. S2b), confirming the previous report showing that C1192G is a high resistance mutation^26^ and our new finding that U1189C mutation is involved in Spc resistance. Growth phenotype of the double mutant (U1189C/C1192G) (Table 3, Supplementary Fig. S2b) was similar to that of the C1192G alone. The high resistance was also reproduced in *E. coli* 16 S rRNA gene with the mutation found in mgSpc2 (C1066U) (MIC >1,024 µg/mL) (Table 3, Supplementary Fig. S2c). As for putative resistance mutations found in mgSpc5, U1183C and U1189C mutations rendered *E. coli* 16 S rRNA highly (MIC = 1024 µg/mL) and modestly (MIC = 256 µg/mL) resistant to Spc, respectively (Table 3, Supplementary Fig. S2c). The MIC of the mutant carrying the double mutations (U1183C/U1189C) was similar to that observed in U1189C individual mutant (MIC = 256 µg/mL) (Table 3, Supplementary Fig. S2c). Some of the resistance mutations (U1189C, C1192G, and G1193A) in the 16 S rRNA gene were moderately disadvantageous for the host *E. coli* MY205, consistent with the previous finding that antibiotic resistance mutations often occur in functionally important sites^7^. (Supplementary Fig. S4).

## Discussion

Investigating the mechanisms of antibiotic resistance has been one of the central issues in the field of ribosomal studies from both clinical and biochemical points of view. However, despite decades of effort, we still know only a little about the diversity of resistance mutations in rRNAs. More precisely, we may not recognize if we know much or little about them. In this study, we developed a general approach to systematically survey resistant rRNA genes/mutations using *E. coli* Δ7, a null mutant of the *rrn* operons. The idea to use the null mutant as a surrogate host organism is based on our recent finding on the functional compatibility of 16 S rRNAs between phylogenetically distant species i.e. 16 S rRNA genes from the Acidobacterial lineage, which were different from *E. coli* at the phylum level, supported the growth of proteobacterial *E. coli* Δ7^20^. We took advantage of this unexpectedly high functional compatibility of 16 S rRNAs in bacteria to characterize the functionality of metagenomic (non-*E. coli*) 16 S rRNA genes in an *E. coli* genetic background, successfully demonstrating the utility of our method by finding novel resistance mutations to Spc, which are supposed to have been thoroughly investigated.

Historically, soon after the clinical use of Spc, lots of studies were undertaken to investigate resistance to the drug. Knowledge obtained in these five decades pointed to Spc inhibiting the translocation step in protein synthesis^21^ by tightly binding to the helix 34 of 16 S rRNAs. Genetic studies revealed that resistance mutations exclusively localized in upper stem of helix 34 in 16 S rRNA^25–34,37^. It is also suggested that Spc indirectly interacts with the ribosomal protein S5^38^; various mutations including point mutations and deletions in S5 confer resistance^39–43^.

As the result of screening a mixed metagenomic library containing 16 S rRNA genes from various environments, we identified four 16 S rRNA genes that rendered the host *E. coli* resistant to Spc, from which five putative resistance mutations (C1066U, U1183C, U1189C, C1192G, and G1193A) were identified (Table 2, Table 4). First, we conducted systematic reverting mutation experiments for these nucleotides, and these experiments generally rendered the metagenomic 16 S rRNA genes susceptible to Spc (Table 4), suggesting that these putative resistance mutations play important roles in rendering these 16 S rRNA genes resistant to Spc. As rRNAs are known to be highly conserved across kingdoms, both in structure and function, particularly for the decoding centre (including Spc binding site) and peptidyl transferase centre, we could study the mechanism of Spc resistance using *E. coli* as the common genetic platform without paying much attention to the species-specific uniqueness of the metagenomic 16 S rRNA genes. In fact, the five putative resistance mutations also rendered *E. coli* 16 S rRNA gene resistant to Spc when each mutation was individually or doubly introduced (Table 3), confirming that they are species-nonspecific resistance mutations. Among them, C1066U, C1192G and G1193A are the known mutations reported in the literature^25–34^. Notably, U1183C and U1189C have not been reported as Spc-resistance mutations; the former is especially interesting because it is the first mutation found in a region other than helix 34 (found in helix 38). Identification of such mutations was unexpected, but ideally coincides with a structural study of the Spc-bound form of the 30 S subunit, which suggested the occurrence of structural rearrangement of the connections between helix 34 and helices 35 and 38^38^. The U1189C mutation had not been described before, but was repetitively identified during our screening (mgSpc1 and mgSpc5) (Table 1). Structurally, both U1183 and U1189 do not interact directly with Spc (Supplementary Fig. S4), which makes it difficult to clearly explain the mechanism behind why mutations in these positions render 16 S rRNA resistant to Spc. The ability to discover such resistance mutations, which are usually difficult to identify from structural data, is certainly one of the merits of our new approach. In addition to the two novel mutations identified in the metagenomic 16 S rRNA genes, we also found that the existence of U in the position 1063 provided non-negligible Spc resistance both on NHMcSpc1 (MIC >1,024 µg/mL) (Table 4) and *E. coli* 16 S rRNA (MIC = 256 µg/mL) (Table 3). C1163 and its base-pairing partner G1193 directly interact with Spc in the crystal structure (Supplementary Fig. S4), which suggests that loss of direct interactions on their mutations renders 16 S rRNA resistant to Spc. Therefore, this C1063U mutation, as well as the other two novel resistance mutations (U1183C and U1189C), have to be added to the list of Spc resistance mutations to monitor the emergence of Spc resistant bacteria.

It should be mentioned, however, that there is one possible insufficiency in this study. Although it is generally true as mentioned above that we do not need to pay much attention about the species-specificities of 16 S rRNA genes (e.g. a specific point mutation can render both *E. coli* and non-*E. coli* 16 S rRNAs Spc resistant equally) in the common genetic background of *E. coli*, there were some cases in which the outcomes of the resistant mutations might be context-dependent. For example, the nucleotide 1189 locating at the upper entrance of h34 conferred a weak resistance to the host *E. coli* upon U to C mutation in *E. coli* 16 S rRNA (MIC = 256 µg/mL) (Table 3). However, reverting mutation in mgSpc1 (C1189U) did not alter the susceptibility and retained high resistance (MIC >1,024 µg/mL) (Table 4). For mgSpc5, however, slight decrease in susceptibility was observed with the same mutation (Table 4), suggesting the existence of sequence (or structure) dependence for a specific resistance mutation toward the expression of Spc resistance. In addition, mgSpc5 showed very high sequence identity with 16 S rRNA from *T. marinus* (1464 of 1472 nucleotides identical) and the strain shares the same “mutations” in the sequence. We tested the Spc susceptibility of the strain but did not find significant resistance (MIC <32 µg/mL). Therefore, although we have effectively identified resistance mutations by genetically reconstituting hybrid 30 S subunits (consist of non-*E. coli* 16 S rRNAs and *E. coli* ribosomal proteins), some resistance of non-*E. coli* 16 S rRNAs may have appeared because of structural perturbation in the “artificial” hybrid ribosome, which effect could be different from “pure” ribosome in native non-*E. coli* bacteria. It is, however, worth noting that this situation could take place after horizontal gene transfer of 16 S rRNA between species^44,45^, suggesting the possibility of a non-canonical scenario for the acquisition of antibiotic resistances.

In this study, we found five resistance mutations to Spc (of which three are novel) in metagenomically-retrieved 16 S rRNA sequences, a traditional and well-studied antibiotic, demonstrating the validity of our experimental approach. The same methodology should be readily applicable to investigate the rRNA-based resistome in clinical samples and other antibiotics. For example, researchers would be able to systematically screen for aminoglycoside resistant 16 S rRNA genes from a faecal sample to estimate the percentage and/or phylogenetic origins of Spc-resistant bacteria in the large intestine at the same time. Another application could be to check the antibiotic susceptibility of a 16 S rRNA gene in a specific pathogenic bacterium that exclusively contains no known specific antibiotic resistance mutations. In such cases, if the 16 S rRNA shows resistance to the antibiotics in *E. coli*, it will strongly suggest the presence of uncharacterized and novel resistance mutation(s) in 16 S rRNA. It would also be interesting to expose environmental or medical samples to a selective pressure of specific antibiotics and select rRNA genes with resistance mutations, from which novel resistance mutations can be found more efficiently. Such experiments would help to better investigate and monitor resistance mutations in rRNAs that have yet to be studied properly.

## Materials and Methods

### Reagents

Ampicillin (Amp), Tmp, and sucrose were purchased from Wako Pure Chemicals. In-Fusion Cloning Kit was purchased from Takara Bio. Lennox LB powder was purchased from Merck. Oligonucleotides were purchased from Sigma Genosys.

### Bacterial strains and growth conditions

*E. coli* MY101 (∆*rrnG* ∆*rrnA* ∆*rrnD* ∆*rrnE* ∆*rrnH* ∆*rrnB* ∆*rrnC*, pMY101, *rna::Km*^R^) is a derivative of SQ171 (∆7 prrn strain)^16,46^, a null mutant of the rRNA (*rrn*) operons in the chromosome. The plasmid pMY101 (*E. coli rrnB*, tRNA^Glu^, tRNA^Asp^, tRNA^Ile^, tRNA^Ala^, tRNA^Trp^, *sacB*, Amp^R^, pSC101 ori) was constructed by transferring the tRNA gene cluster encoded by pTRNA67^16^ into pRB101^47^ at the site between the 16 S and 23 S rRNA genes. The resultant pMY101 plasmid contains the entire *E. coli rrnB* operon, and complements the growth of MY101. The strain was cultured at 37 °C in LB (1% [w/v] tryptone, 0.5% [w/v] yeast extract, 0.5% [w/v] NaCl) medium containing 100 µg/mL Amp. *E. coli* MY205 is a derivative of MY101, in which pMY101 was completely replaced with pMY205mPAG2 (*E. coli rrnB*, tRNA^Glu^, tRNA^Asp^, tRNA^Ile^, tRNA^Ala^, tRNA^Trp^, Tmp^R^, p15a ori) using sucrose-induced counter-selection. The pMY205mPAG2 plasmid was used as a vector to introduce foreign 16 S rRNA genes. The MY205 strain was cultured in LB medium containing 10 µg/mL Tmp at 37 °C. *Thalassolituus marinus* (NBRC 107590) was obtained from NITE Biological Resource Centre (Japan). It was grown in LB medium in the presence or absence of 40 µg/mL Spc at 25 °C.

### Constructing and screening a metagenomic library of 16 S rRNA genes for Spc resistance

Environmental metagenomes were purified from various sources including fermentation products^48^, wood composts^49^, and activated sludge^50^, as described previously^24^. These metagenomic samples were mixed and used as the source material of the various 16 S rRNAs. The 16 S rRNA gene fragments were PCR-amplified from the metagenomic DNA as described^24^. Briefly, a set of primers Bac1f (5′-AAATTGAAGAGTTTGATC-3′) and UN1542r (5′-TAAGGAGGTGATCCA-3′) were used to amplify the full-length of the 16 S rRNA genes, which was replaced with the *E. coli* 16 S rRNA gene in pMY205mPAG2. To this effect, the vector was inversely amplified using another set of primers Bac1r (5′-GATCAAACTCTTCAATTTAAAAGTTTGACGCTCAAAG-3′) and UN1542f (5′-TGGATCACCTCCTTACCTTAAAGAAGCGT-3′)^24^. Equimolar vector and insert fragments were combined and ligated by incubation at 50 °C for 1 h using the In-Fusion Cloning Kit. The reaction mixture was introduced into *E. coli* JM109 and the colonies were grown on LB/Tmp agar plates at 37 °C. Plasmids were extracted from the pooled colonies (approximately 10,000) and used to transform the *E. coli* Δ7 strain MY101. We then recovered the colonies from the LB/Tmp agar plates (approximately 10,000), resuspended them in LB broth, appropriately diluted and spread the broth on LB/Tmp agar plates containing 5% (w/v) sucrose. After counter-selection on sucrose-containing plates to eliminate pMY101, we obtained approximately 2,000 colonies. The MY205 library was then screened on LB/Tmp agar plates containing 40 µg/mL Spc. MY205 carrying *E. coli* 16 S rRNA (as the Spc sensitive control) was constructed using pMY205mPAG2 (containing a *rrnB* operon) without mutations.

### Antibiotic resistance test

MY205 derivatives were grown overnight in LB/Tmp at 37 °C. The saturated culture was then diluted at a concentration of 1/1,000 in LB/Tmp. The diluted culture (1 µL) was then inoculated into 200 µL of LB/Tmp containing varied concentrations (0–1,024 µg/mL) of Spc in a flat-bottomed 96-well plate. The plate was then incubated with vigorous agitation (9.2 Hz) on a Sunrise Thermo RC-R plate reader (Tecan) at 37 °C and the OD_600_ was continuously monitored every 15 min, without reducing the baseline value of the negative control in which bacteria was not inoculated into the medium. The minimum inhibitory concentration (MIC) for each MY205 derivative was defined as the minimal concentration of Spc which OD_600_ value after 10 h-cultivation did not exceed 0.15, close to the baseline value (0.13).

### DNA sequencing and analyses

DNA sequencing was carried out using the Sanger method with an Applied Biosystems automatic DNA sequencer (ABI PRISM 3130xl Genetic Analyzer) and an Applied Biosystems BigDye (ver. 3.1) Kit. BLAST search^51^ was carried out on 15 August, 2017 using the NCBI nucleotide database “16 S rRNA sequences (Bacteria and Archaea)” with the program selection optimized for “Highly similar sequences (MegaBLAST)”. Multiple sequence alignment was performed using the MAFFT v7 program^52^.

### Data and materials availability

DNA sequence data reported in this study have been deposited under the accession numbers, LC306679 (mgSpc1), LC306680 (mgSpc2), LC306681 (mgSpc5), and LC306682 (NHMcSpc1), respectively.

## Electronic supplementary material

Supplementary Figures 1–4
