# Supplementary material for: Functional metagenomic approach to identify overlooked antibiotic resistance mutations in bacterial rRNA
Source: Sci Rep. 2018 Apr 3;8:5179. doi: 10.1038/s41598-018-23474-4 (PMC5882664; doi:10.1038/s41598-018-23474-4)
Supplement: Supplementary file 1 — Supplementary Figures 1–4 [file 41598_2018_23474_MOESM1_ESM.pdf]

Supplementary Materials

Functional metagenomic approach to identify overlooked antibiotic resistance mutations in bacterial rRNA

Kentaro Miyazaki and Kei Kitahara

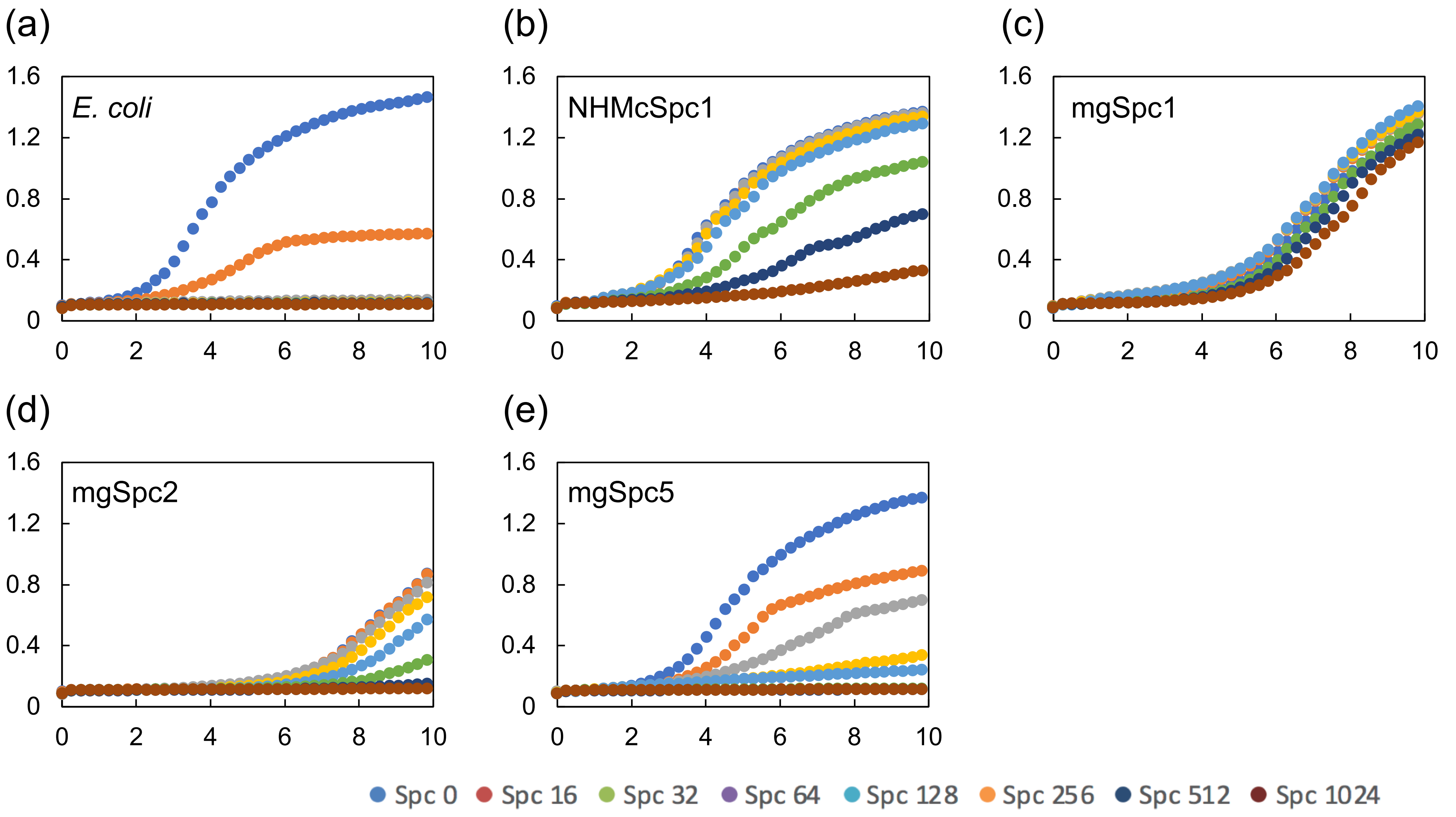

Supplementary Figure S1. Growth curves of *E. coli* Δ7 (MY205) carrying the wild-type and spectinomycin resistant 16S rRNA genes obtained from the metagenome. (**a**) *E. coli* 16S rRNA, (**b**) NHMcSpc1, (**c**) mgSpc1, (**d**) mgSpc2, (**e**) mgSpc5. Cells were grown and their OD_{600} values were monitored every 15 min without reducing the baseline value for the negative control (0.13) in which bacteria was not inoculated to the medium. X-axis, time (h); Y-axis, OD_{600}. Growth curves are differently coloured depending on the concentrations of spectinomycin added to each medium.

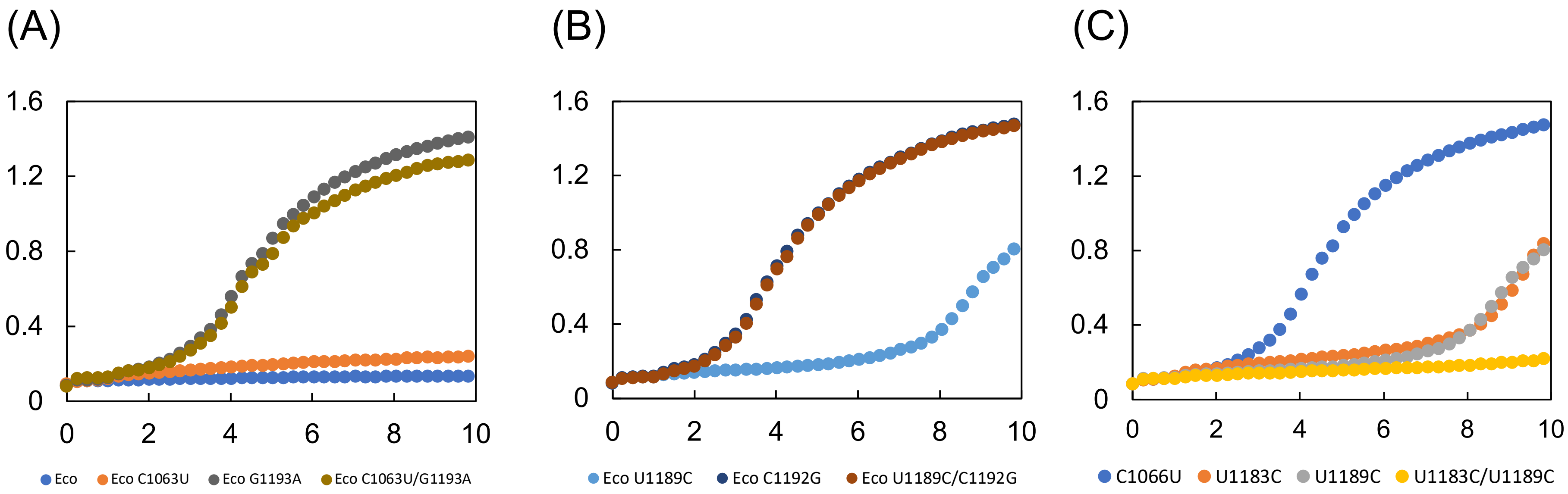

Supplementary Figure S2. Growth curves of *E. coli* Δ7 (MY205) carrying the wild-type and mutant *E. coli* 16S rRNA genes in the presence of 32 µg/mL spectinomycin. (**a**) Wild-type, C1063U, G1193A, and C1063U/G1193A (**b**) U1189C, C1192G, and U1189C/C1192G (**C**) C1066U, U1183C, U1189C, and U1183C/U1189C. Cells were grown and their OD_{600} values were monitored every 15 min without reducing the baseline value for the negative control (0.13) in which bacteria was not inoculated to the medium. . X-axis, Time (h); Y-axis, OD_{600}. Growth curves are differently coloured depending on the mutations introduced.

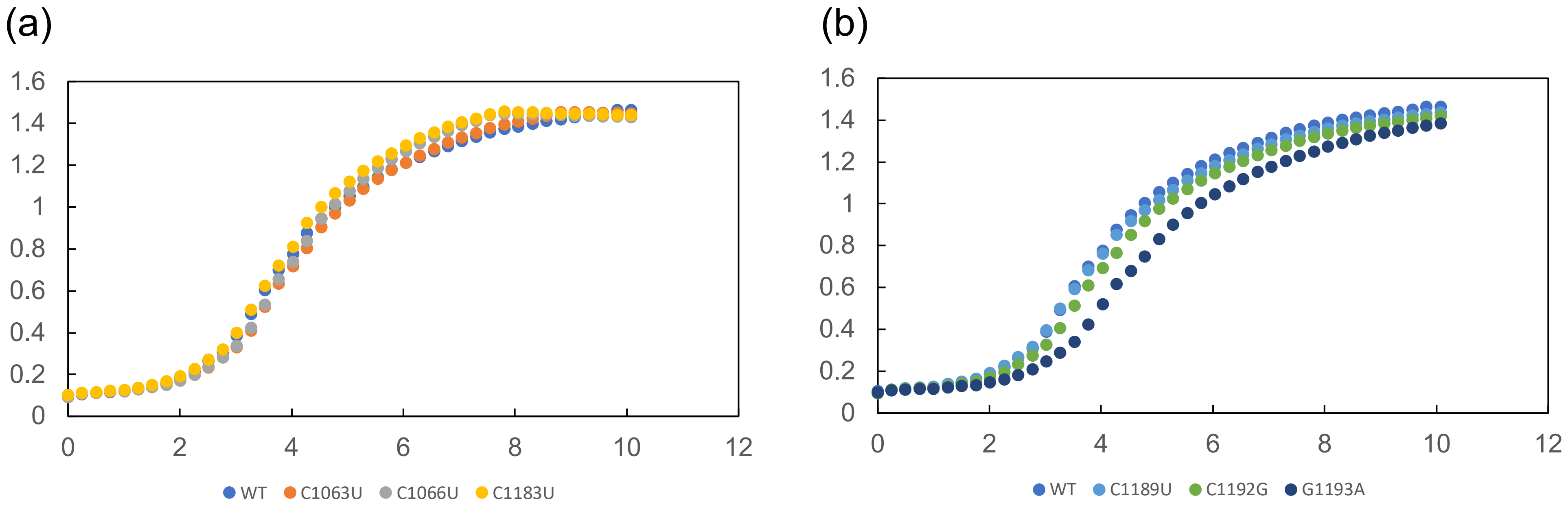

Supplementary Figure S3. Growth curves of *E. coli* Δ7 (MY205) carrying the wild-type and mutant *E. coli* 16S rRNA genes in the absence of spectinomycin. (a) Mutations that did not affect cell growth. (b) Mutations that diminished cell growth. Cells were grown and their OD_{600} values were monitored every 15 min without reducing the baseline value for the negative control (0.13), in which bacteria were not inoculated into the medium. X-axis, Time (h); Y-axis, OD_{600}. Different colours in the growth curves indicate different mutations introduced

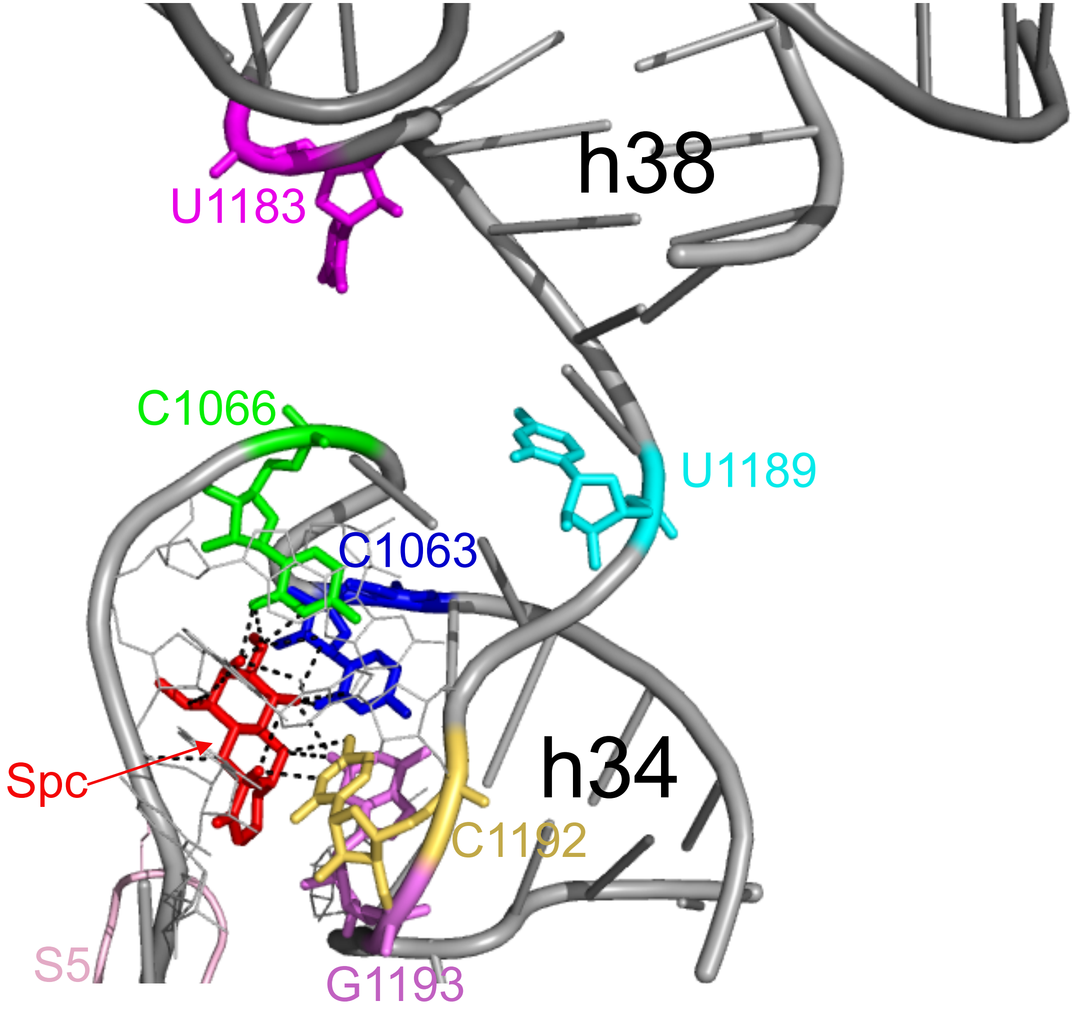

Supplementary Figure S4. Nucleotides whose mutations render 16S rRNA resistant to spectinomycin (Spc). C1063, C1066, U1183, U1189, C1192 and G1193 in 16S rRNA were mapped onto the crystal structure of the *E. coli* 30S ribosome subunit with Spc (PDB:2QOU). The Spc-binding site of the ribosomal protein S5 is also shown. Hydrogen bonds between Spc and nucleotides or amino acids are shown as dotted black lines. C1063, C1066, C1192, and G1193 show direct interactions with Spc via hydrogen bonds. Although U1183 and U1189 exist relatively in the vicinity of the Spc-binding site, they do not interact with Spc directly.
